# Statistical Distribution Analysis of Navigation Positioning System Errors—Issue of the Empirical Sample Size

**DOI:** 10.3390/s20247144

**Published:** 2020-12-13

**Authors:** Mariusz Specht

**Affiliations:** Department of Transport and Logistics, Gdynia Maritime University, Morska 81-87, 81-225 Gdynia, Poland; m.specht@wn.umg.edu.pl

**Keywords:** statistical distribution, navigation positioning system, position error, sample size, Position Random Walk (PRW), Global Positioning System (GPS), Differential Global Positioning System (DGPS), European Geostationary Navigation Overlay Service (EGNOS), Decca Navigator system

## Abstract

Positioning systems are used to determine position coordinates in navigation (air, land, and marine). Statistical analysis of their accuracy assumes that the position errors (latitude—*δ_φ_* and longitude—*δ_λ_*) are random and that their distributions are consistent with the normal distribution. However, in practice, these errors do not appear in a random way, since the position determination in navigation systems is done with an iterative method. It causes so-called “*Position Random Walk*”, similar to the term “*Random Walk*” known from statistics. It results in the empirical distribution of *δ_φ_* and *δ_λ_* being inconsistent with the normal distribution, even for samples of up to several thousand measurements. This phenomenon results in a significant overestimation of the accuracy of position determination calculated from such a short series of measurements, causing these tests to lose their representativeness. This paper attempts to determine the length of a measurement session (number of measurements) that is representative of the positioning system. This will be a measurement session of such a length that the position error statistics (*δ_φ_* and *δ_λ_*) represented by the standard deviation values are close to the real values and the calculated mean values ($\bar{\varphi }$ and $\bar{\lambda }$) are also close to the real values. Special attention will also be paid to the selection of an appropriate (statistically reliable) number of measurements to be tested statistically to verify the hypothesis that the *δ_φ_* and *δ_λ_* distributions are consistent with the normal distribution. Empirical measurement data are taken from different positioning systems: Global Positioning System (GPS) (168′286 fixes), Differential Global Positioning System (DGPS) (864′000 fixes), European Geostationary Navigation Overlay Service (EGNOS) (928′492 fixes), and Decca Navigator system (4052 fixes). The analyses showed that all researched positioning systems (GPS, DGPS, EGNOS and Decca Navigator) are characterized by the Position Random Walk (PRW), which resulted in that the empirical distribution of *δ_φ_* and *δ_λ_* being inconsistent with the normal distribution. The size of the PRW depends on the nominal accuracy of position determination by the system. It was found that measurement sessions consisting of 1000 fixes (for the GPS system) overestimate the accuracy analysis results by 109.1% and cannot be considered representative. Furthermore, when analyzing the results of long measurement campaigns (GPS and DGPS), it was found that the representative length of the measurement session differs for each positioning system and should be determined for each of them individually.

## 1. Introduction

Navigation is one of the technical sciences where statistical elements are used. It is most often defined as a field of study that focuses on the process of monitoring and controlling the movement of a craft or vehicle from one place to another [1]. A similar, but a more extended definition of navigation is given by Bernhard Hofmann-Wellenhof, who states that navigation—deals with moving objects (mostly vehicles) and involves trajectory determination and guidance [2]. Cezary Specht goes even further [3], stating that navigation is the process of controlling the movement of an object and he extends the process to include living beings. It is beyond any doubt that the field of navigation includes four general categories: land navigation, marine navigation, aeronautic navigation, and space navigation, as well as that navigation usually refers to three-dimensional objects or vehicles, whereas positioning is typically applied to point determination [2]. To the contrary, the International Association of Lighthouse Authorities (IALA) in its definition stresses the role of positioning, stating that navigation is the art or science of determining the position and course of a ship or aircraft by means of observations on board, whereby difficulties and dangers are avoided and the desired destination is reached as quickly and safely as possible [4].

In navigation, as in many other scientific disciplines, the subject of statistical analysis is not fundamental knowledge, which is probably why a dedicated publication considering, in a wide way, its various aspects has not yet appeared. Navigation, as a technical science, uses statistical methods similar to other disciplines of this type. In addition, it should be stressed that issues relating to probability calculus are found in navigation in a rather limited form.

Fundamental, international publications on navigation, in particular, Nathaniel Bowditch’s “*American Practical Navigator*”, regularly published since 1802 [1], as well as other book items [2,5], only discuss individual elements of statistics. Specifically, they can be found in chapters related to the mathematical basis of positioning and the evaluation of its accuracy. It should be noted that since the navigation process can only be carried out in real-time, it seems that, apart from assessing the positioning accuracy to determine errors by statistical methods, no statistical analyses are used in practical navigation (when controlling an object). Scientific research in navigation presents yet another issue with statistical methods covering all relevant aspects of navigation, such as communication, satellite [6,7] and terrestrial positioning methods [8], collision avoidance, traffic surveillance (aircrafts, vehicles, and vessels) and measurement systems [9,10,11], as well as navigation equipment [12,13].

**Figure 1:**
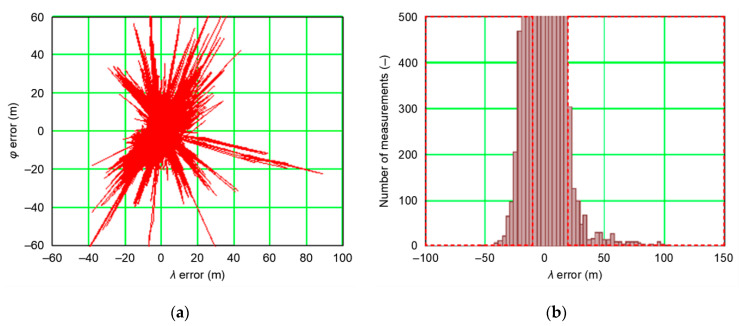
(**a**) European Geostationary Navigation Overlay Service (EGNOS) position error distribution (1′774′705 fixes); (**b**) histogram in relation to the *λ* with outliers marked in red during the measurement campaign in 2006 [18].

The assumption that a position line error in navigation has a normal distribution is commonplace for book authors [2,5,14], as well as in monographs, regulations, and standards directly related to the statistical analysis of position errors [15,16], which is relatively simple due to the close relationship between population and probability, based on the determination of standard deviation. However, it is common for scientific publications on position errors in navigation systems to avoid questioning the truth of the assumption on the normality of the distribution of empirical data. If it turns out that measurement data (position errors of navigation systems) significantly deviate from the normal distribution, then the measurement results given in the form of Root Mean Square (RMS) and Distance Root Mean Square (DRMS), are false. This issue was signaled in individual publications [16,17]. This hypothesis can be exemplified by European Geostationary Navigation Overlay Service (EGNOS) system measurements carried out in 2006 and covering some 2 million fixes. The distribution of position errors shown in Figure 1 proves undisputedly that these measurements are not consistent with the chi-square distribution, being the sum of the two normal distributions (*δ_φ_* and *δ_λ_*), as indicated by numerous gross errors and outliers.

The example distribution of EGNOS position errors from 2006 (Figure 1) proves that the commonly accepted a priori hypothesis that position errors of navigation systems always have the normal (1D) or chi-square (2D) distribution is not obvious. Therefore, before calculating position errors (1D and 2D), a method consisting in determining RMS is most often used in navigation and it is thus reasonable to test the consistency of empirical data (position errors) with the normal distribution to avoid gross errors. Testing should be based on commonly used statistical tests with Anderson–Darling and Kolmogorov–Smirnov tests being the most frequently applied. However, it should be stressed that for large samples (several hundred thousand measurements), it is practically inevitable that outliers appear, which will cause the thesis on the sample consistency with the normal distribution to be verified negatively, which in turn will lead to the false assumption that measurement errors do not follow Gauss’s statistics. Therefore, proper selection of the number of tested measurements (position errors), which will ensure high reliability for concluding the consistency of empirical data with the theoretical distribution (normal or chi-square) is of key importance even if not yet described in the navigation literature.

Studying statistical distributions of each random variable requires a thorough knowledge of the statistical nature of the analyzed value, because without it the use of specific statistical methods may lead to wrong conclusions. In most experimental research, regardless of the studied phenomena, it is assumed that the distribution of their errors is similar to normal. In this context, positioning in navigation systems is exceptional. The main problem to be discussed here is the Position Random Walk (PRW). It consists in the fact that in navigation systems subsequent positions of the stationary receiver do not appear at random, i.e., as a result of “*jumping*” around the mean, but they always appear near the previous position. Based on this, it cannot be stated that a random variable (*δ_φ_* or *δ_λ_*) corresponds to the basic characteristic of a normal distribution, i.e., the absence of correlation between measurements. There are three main reasons for this issue:

- The iterative method used to determine coordinates, where the next position is calculated from the previous one.
- Widespread use of Kalman filtering in positioning system receivers for filtering out movement (course and speed) parameters.
- Normally navigation positioning system errors are correlated over time.

**Figure 2:**
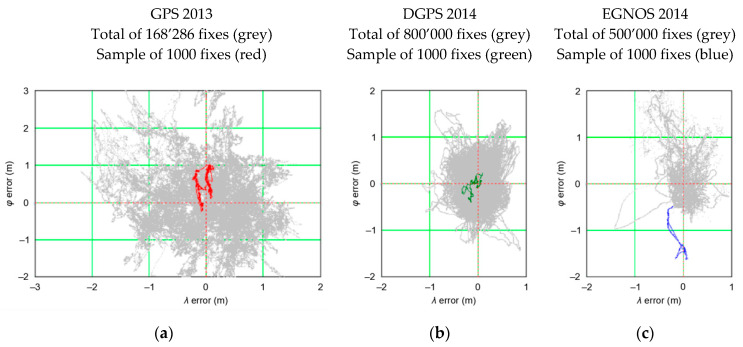
Examples of the Position Random Walk (PRW) for (**a**) Global Positioning System (GPS); (**b**) Differential Global Positioning System (DGPS); (**c**) EGNOS.

The term PRW is not known in the navigation literature. In my opinion, its very essence resembles a “*Random Walk*” as defined by Carl Pearson in 1905 [19], even though it is, in fact, very different since a finite set of positions that may result from subsequent iteration cannot be determined. According to Pearson, the Random Walk process most often refers to a specific category of Markov chains or Markov processes that cannot be applied to the positioning problems under consideration. However, the common feature of the process described by Pearson and the process of determining position in navigation systems is the Random Walk of position coordinates, hence this term was introduced into the analyses. Its essence is best reflected in Figure 2, which presents the position error distribution of three different navigation systems recorded by receivers during stationary measurements (no movement). These are Global Positioning System (GPS), Differential Global Positioning System (DGPS), and EGNOS systems.

The three examples of PRW shown in Figure 2 clearly indicate that the coordinates determined by the receiver move along theoretical (based on the current state of knowledge), unpredictable path. Hence, the proposed Position Random Walk term, which describes this phenomenon, seems adequate. It should be noted that the position coordinates do not jump randomly around the mean coordinate values (0, 0), as one would expect if *φ* and *λ* errors appear randomly.

The PRW as shown in Figure 2 proves that if we were to assess the position error based on a sample of only 1000 measurements, it would turn out that the result would be far from being representative of the entire population (grey). This raises the obvious question of the sample length required for the results (position error, $\bar{\varphi }$ and $\bar{\lambda }$) to conform with the actual values, which in this paper are the actual accuracy values of the positioning systems or the results determined based on a very long measurement campaign.

The aim of the article is:

- To develop a method for calculating the length of a representative sample of any positioning system, the results of which would be considered to be close to the actual accuracy values of the positioning system.
- To propose a method for assessing the consistency of navigation system position errors with theoretical statistical distributions (normal distribution), based on empirical measurement data, which will ensure high reliability assessment with a high statistical test power.

The publication has been divided into four parts. The Materials and Methods section describes in detail the normal distribution in positioning. The first subsection ends with four research questions regarding, among others, minimum number of measurements (referred to in statistics as representative sample) for statistical testing of *φ* and *λ* errors, as well as consistency of the empirical distributions of these errors with the normal distribution for different navigation positioning systems. This section also contains information on methods of statistical testing consistency of empirical distributions with theoretical methods, which were subsequently used to assess the distributions of position errors of an example navigation system (GPS). Verification of statistical hypotheses, with particular focus on the concept of “*effect size*” represented by the Cohen’s d, was then conducted. This has a significant impact on the minimum length of the measurement session which ensures the representativeness of the tested population. The last subsection discusses the selection of the optimal number of navigation system position errors based on an evaluation of the statistical test power. The Results section begins with an analysis of a phenomenon, which the author calls “*Position Random Walk*”. This is a unique feature of positioning systems, where each subsequent position is in the direct neighborhood of the previous one. Thanks to this, *φ* and *λ* errors (with a small number of measurements) are not normally distributed and the mean value ($\bar{\varphi }$ and $\bar{\lambda }$) and RMS error deviate from the actual values. A GPS measurement campaign was then conducted based on which a method for calculating the minimum length of a representative session and the minimum number of measurements to be tested to verify consistency with the normal distribution was proposed. The paper ends with a discussion and conclusions.

The article begins a series of monothematic publications, the aim of which will be statistical distribution analysis of navigation positioning system errors.

## 2. Materials and Methods

### 2.1. Normal Distribution in Positioning

Major arguments justifying the use of normal distribution in research on positioning in navigation include [20]: intuition and tradition, simplicity of the distribution [21], consistency with the central limit theorem, as well as it is almost always applied as an approximation [22].

In navigation calculation of position coordinates require determining two position lines (2D) or three position areas (3D). The measured value (*φ*, *λ* or altitude from the reference level) *x* is a random variable with probability density function *f(x)*, mean value *μ* and variance *σ^2^* described by the relationship:

(1) $$\mu =\int_{-\infty }^{+\infty }x\cdot f\left(x\right)dx$$

(2) $$\sigma^{2}=\int_{-\infty }^{+\infty }{\left(x-\mu \right)}^{2}\cdot f\left(x\right)dx$$

Standard deviation *σ* is the square root of the variance, which may be transposed to a standardized random variable *z* as:

(3) $$z=\frac{x-\mu }{\sigma }$$

With zero mean and unit variance, the Gauss probability density function expressed by the formula:

(4) $$f\left(z\right)=\frac{1}{\sqrt{2\pi }}e^{\frac{-z^{2}}{2}}$$

For which probability is calculated from:

(5) $$P\left(-\alpha <z<\alpha \right)=\int_{-\alpha }^{\alpha }f\left(z\right)dz=\frac{1}{\sqrt{2\pi }}\int_{-\alpha }^{\alpha }e^{\frac{-z^{2}}{2}}dz$$

where the standardized random variable *z* takes any value in the symmetric interval (−α,α).

**Figure 3:**
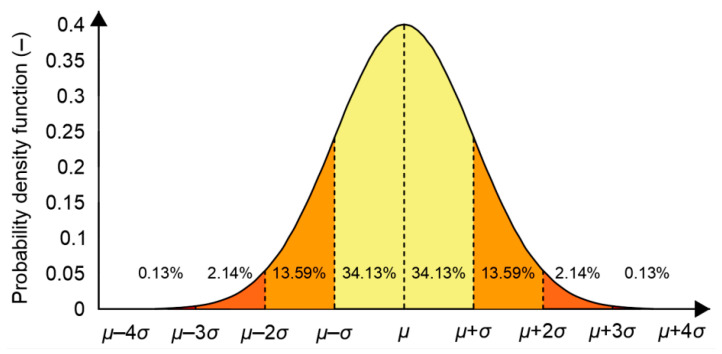
Normal probability density function.

The Gauss distribution is also commonly called the “*normal distribution*” and is often described as a “*bell-shaped curve*” (Figure 3) and is used in the analysis of position errors, which should be emphasized, separately from each of the measured coordinates (*φ*, *λ* or altitude from the reference level).

Position coordinates (2D) constitute a superposition of the errors of two independent normal distributions (*φ* and *λ*). Therefore, let us examine the error in determining a 2D position, where the statistical distributions of measurement errors of two single position lines are consistent with the normal distribution. 2DRMS is the measure of 2D position error. It means “*two-dimensional RMS*”. 2DRMS usually stands for “*twice distance RMS*” in which the “*distance*” is measured in a two-dimensional space, the horizontal plane [16]. The random variable *μ* is no longer normally distributed but follows the chi-square distribution with the probability [2]:

(6) $$P\left(0<\mu <\alpha \right)=1-e^{\frac{-\alpha }{2}}$$

2DRMS is now the more commonly used term and is often seen within previous Federal Radionavigation Plans (FRP). It refers to twice the distance root mean square and not to the two-dimensional RMS. To compute the DRMS from a set of data it is simply necessary to compute the root mean square of the radial errors, i.e., the linear distances between the measure and known (or mean) positions, and double the result. A disadvantage of the 2DRMS measure is that does not have a constant probability attached to it. Essentially the associated probability is a function of the ellipticity of the relevant error ellipse resulting from a particular satellite geometry. On the assumption that the pseudorange errors are normally distributed, this probability will be in the range of 95.4% to 98.2%. Table 1 summarizes the numerical relationship between the population and probability.

From the analysis presented, it is clear that the use of RMS (for *φ* and *λ* separately) or 2DRMS commonly accepted in navigation for the 2D position requires that navigation system position errors follow the normal distribution. To confirm this assumption, it is necessary to correctly carry out statistical tests of a specific population of the real (measured) *φ* or *λ* errors of a positioning system, which will ensure normality of the distributions. However, the subject literature lacks concrete examples of calculations and tests performed on empirical data from positioning systems that would indicate which measurement sample should be considered representative, what number of measurements should be statistically tested, which statistical test to use and, most importantly, whether or not there are any particular features of the positioning process implemented by navigation systems that make it necessary to use the classical approach for testing position error distributions well-known from general statistics. There are four main questions to be posed in this context and these are also the subject of this publication:

1. To correctly determine the accuracy of position determination (described by RMS and 2DRMS measures) using the navigation system, can any number of measurements be taken, followed by determination of the standard deviation and the mean values of *φ* and *λ* (which are most often assumed to be real), or is there any minimum number of measurements (referred to in statistics as representative sample) that is necessary to ensure that its RMS and mean values of *φ* and *λ* are identical or very close to the true accuracy of the positioning system?
2. Do the statistical distributions of empirical *φ* and *λ* errors of the positioning system conform with the normal distribution for any sample size (100, 1000, or 10′000 measurements, etc.)?
3. Are the answers to questions 1 and 2 different depending on the type of positioning system (GPS, GLONASS (GLObal NAvigation Satellite System), DGPS, EGNOS, etc.)?
4. Are there any special statistical features of the distributions of navigation system position errors that distinguish them from other, typical studies of empirical distributions in technical or other sciences?

These are questions about the fundamental positioning issues in navigation, but despite the magnitude of the problem, no answers to the above questions could be found in the international literature.

### 2.2. Statistical Testing of the Empirical Distribution of Position Errors

**Figure 4:**
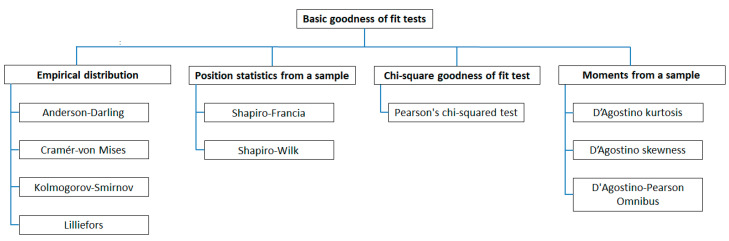
Division of basic statistical tests used to examine the consistency of empirical distribution with the theoretical normal distribution. Own study based on: [23].

In order to be able to express the accuracy of positioning systems in navigation using commonly available and valid measures (RMS or 2DRMS), it is necessary to ensure that position errors determined separately for *φ* and *λ* are normally distributed and the length of the measurement session provides that the results are representative. The basic method of establishing this consistency is to test the fit between distributions of empirical position errors and theoretical distributions. Statistical testing should be used for this purpose. As described in [23], statistical testing can be divided into (Figure 4):

- Tests based on empirical distributions: Anderson–Darling [24], Cramér-von Mises [25,26], Kolmogorov–Smirnov [27,28], and Lilliefors tests [29].
- Tests based on position statistics from a sample: Shapiro–Francia [30] and Shapiro–Wilk tests [31].
- Tests based on the chi-square goodness of fit test: Pearson’s chi-squared test [32].
- Tests based on moments from a sample: D’Agostino kurtosis [33], D’Agostino skewness [34], and D’Agostino–Pearson Omnibus tests [35].

When studying statistical distributions of position errors in navigation, we are interested in testing fit of 1D errors (*φ* and *λ*) with a family of normal distributions and fit of 2D errors with the chi-square distribution. To this end, statistical hypotheses are verified, which means that any judgement on the population issued without detailed examination and verification is now tested. These allow determining whether the results obtained for the sample can be applied to the whole population [36].

For statistical testing, an alternative hypothesis (*H*_1_) is first formulated, which is identical to the verified hypothesis (e.g., the distribution of *φ* errors fits normal distribution with the following parameters: *μ* and *σ*). This is followed by the null hypothesis (*H*_0_), being its inverse [37]. For verifying the correctness of hypotheses made, it is attempted to reject the *H*_0_ hypothesis as unlikely. The confidence with which this verification is made is determined by the arbitrarily accepted significance level (*α*). It is defined as the arbitrarily accepted risk that the null hypothesis considered to be false is indeed true. This allows the level of which deviations observed in the sample above the test will result in accepting the *H*_1_ hypothesis to be determined. The choice of the *α* value is up to the researcher, it depends on the nature of the problem (distribution of 1D and 2D position errors) and how accurately the hypotheses will be verified. In practice, α = 0.05, sometimes 0.1 or 0.01 (and in special individual cases 0.001) are usually used in natural sciences. Significance levels higher than 0.1 and lower than 0.001 are not used in practice. A small *α* value is essential for testing because it prevents false confirmation of false test results (conclusions), formulated in the form of the alternative hypothesis.

The following hypotheses should be formulated for navigation issues:

- *H*_0_—the empirical distribution of *φ* or *λ* errors of the *X* positioning system fits the normal distribution.
- *H*_1_—the empirical distribution of *φ* or *λ* errors of the *X* positioning system does not fit the normal distribution.

Analogous hypotheses on the fit of 2D position error distributions can be formulated with respect to the chi-square distribution.

Please note that the significance level α is equal to the probability of making a type I error. A type I error (false positive) consists of rejecting the *H*_0_ hypothesis, which is actually not false. The estimation of the probability of making a type I error is designated with the symbol α. Another error is the so-called false negative also referred to as type II error. Type II error (false negative) consists of not rejecting the null hypothesis when it is actually false. The estimation of the probability of making a type II error is designated with the symbol *β*, and its complement is called the “*test power*”. Statistical power is the probability of not making a type II error. The greater this probability, the better the test as a tool for distinguishing between true and false hypotheses. Power can be expressed as a complement to the probability of making a type II error (*β*), i.e., 1-*β* (Table 2).

The magnitude of these errors is a certain probability. Obviously, it would be best if both errors were minimized at the same time. However, this is impossible. Therefore, a certain compromise is sought between these errors by constructing the so-called most powerful tests, i.e., tests which, with a fixed probability of committing a type I error, minimize the probability of type II error. If the test has high power (its value approaches one), it is easier to reject the *H*_0_ hypothesis, but it is also easier to make a type I error. However, if the test is weak, it is more difficult to reject the null hypothesis and it is easier to make a type II error. As can be seen, for testing the hypotheses are not treated symmetrically. We could say that the test is constructed in such a way as to provide *H*_0_ hypothesis unless the sample supports *H*_1_ hypothesis strongly enough. If the null hypothesis is rejected, it follows that the probability of making a type I error is less than α. However, if the test fails to reject *H*_0_ hypothesis, then a type II error is possible. Tests which only take type I errors into consideration are called significance tests and the probability of type I errors is determined with a significance level. In these tests, the type II error is completely ignored (the probability of making it is indicated by *β*). Therefore, in such tests there is no talk of accepting the null hypothesis, but only about the lack of grounds for rejecting it [38].

### 2.3. Effect Size as a Function of Measurement Errors

Correct selection of power of the chosen test and planning of the sample size (number of measurements) require a pre-assumption of effect size. Further calculations are made on this basis (sample size and α). The effect size is a quantitative measure of the strength of a given phenomenon, for example, the difference between a control group (theoretical position error statistics) and an experimental group (experimental position error statistics) calculated from data [39]. The effect size value is a number calculated from data based on a specific mathematical formula describing the magnitude of the effect size (e.g., variability). In positioning, it plays an important role because it allows the correct assessment of representative sample size and the number of measurements that will be tested for fit with the normal distribution.

In order to obtain the desired test power for given sample size, the formulas developed by Jacob Cohen presenting the relationship between effect size and power for samples of varying sizes are often used [40]. Cohen’s definition of effect refers directly to a measurement, where it means an estimation of the extent to which the effect is present in a population [41]. He proposed using the difference between the mean measured value determined based on empirical data ${\bar{\mu }}_{E}$ and the actual value *μ_T_*, divided by the value of the population’s standard deviation *δ_E_* according to the following formula:

(7) $$d_{COHEN}=\frac{\left|{\bar{\mu }}_{E}-\mu_{T}\right|}{\delta_{E}}$$

where

*d_COHEN_*—measure of the Cohen’s effect;

${\bar{\mu }}_{E}$—mean value determined based on empirical data (*φ* or *λ* obtained from measurements);

*μ_T_*—actual value of the measured variable. For position measurements, this is a value determined in surveys, as well as $\bar{\varphi }$ or $\bar{\lambda }$ measurements are also often accepted;

*δ_E_*—population standard deviation.

Jacob Cohen proposed that 0.2, 0.5, and 0.8 be defined as small, medium, and large effect, respectively. The assessment of effect size defined by Cohen has been widely accepted by other researchers [41,42].

**Figure 5:**
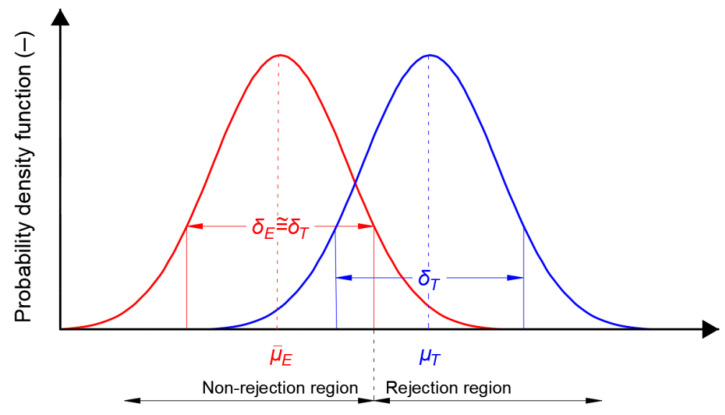
Relationship between the empirical (red) and the actual (blue) probability density function of measurement errors, together with the rejection and non-rejection regions of the distribution fit hypothesis.

In illustrating the effect size in relation to position errors in navigation systems, the actual statistical distribution of *φ* or *λ* errors is shown in blue in Figure 5. This can be obtained by calculating the actual *φ* or *λ* parameters and determining the RMS values. Mean *φ* or *λ* can be obtained using surveying methods (e.g., Real Time Kinematic (RTK) or Real Time Network (RTN) with an error of about 1 cm (*p* = 0.95)) or determined as a mean from a very large population of measurements. The statistical distribution of empirical results from the measurements is marked in red. Please note that this sample can be considered representative if its mean value is close to the real one and the value of standard deviation (measurement errors) is also similar to the real one. Otherwise a sample should be considered as unrepresentative. This fact is material for the analyses carried out in the Results section. It will be crucial for further considerations of the distributions of position errors in navigation systems. Moreover, it should be noted that according to the theory of [43] in many precise positioning applications, such as RTK or RTN, the underlying model of satellite positioning is of the mixed-integer type, as a result of which the estimated position errors will not have a normal distribution, but instead a multi-modal distribution.

### 2.4. Power Test and Sample Size

Correct selection of the number of navigation system position error measurements is of key importance in assessing the fit between the empirical and the theoretical distribution. In navigation, for a large sample (more than 10′000 measurements) accepting them all for analysis can very often result in rejection of the hypothesis on consistency with a normal distribution, even though a significant part of the measurements is normally distributed. Therefore, in statistical testing appropriate choice of power (1−*β*) is important and it depends on:

- Actual effect size against the background of random variation in the population.
- Assumed significance level α (usually 0.05).
- Sample size *n* used in the test.

Depending on research type, different desirable levels of this indicator are adopted. For example, based on articles from some leading psychological journals [44,45,46], or studies published in medical journals [47], the median power is about 0.45 [48]. In line with Jacob Cohen’s recommendations [41], the desired level for this indicator is 0.8 and the desired significance level is *α* = 0.05 [42,49]. Therefore, it seems justified to accept both of these values for testing position errors (1D and 2D).

The sample size is another factor to be estimated when selecting the test power. According to [48], the larger the sample size, the smaller the standard deviation *δ_E_*. With increasing sample size, the common area between the actual and hypothetical distribution of the tested variable decreases. As a result, the probability of selecting a sample size sufficient to reject the false null hypothesis increases. Therefore, with unchanged values of other parameters, the larger the sample size, the smaller the standard error of the mean and the greater the test power. A very important point must be made here. This thesis is true in all statistical tests, where measurement error has a fully random distribution. The problem is that this is not the case with positioning using navigation systems. This issue will be described in detail in the Position Random Walk Analysis subsection of the Results section.

**Figure 6:**
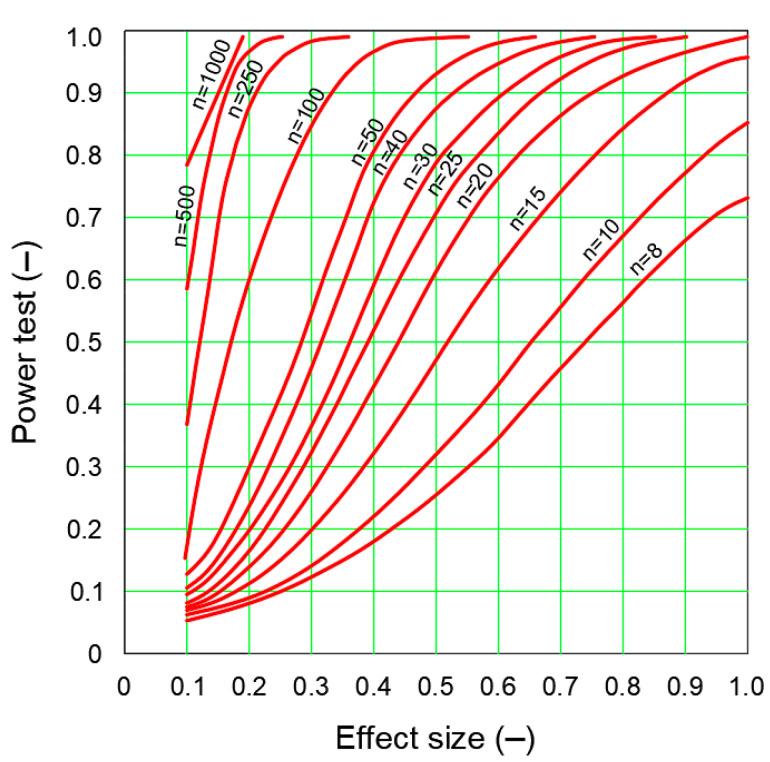
Examples of test power curves. Own study based on: [48].

Once the factors influencing power (*d_COHEN_*, *n*, test selection, *α* and *δ_E_*,) have been calculated, the desired power level can be determined for a sample size adequate to the minimum effect size [48]. Diagrams with power curves can be used for this purpose (Figure 6) [41].

When determining the test power, a test power diagram should initially be selected for set significance level and test type. Once the appropriate power curves have been selected, the desired effect size (the effect large enough is assumed to be 0.8) and the test power (it is recommended to be 0.8) are defined. As mentioned in [50], it should be noted that each scientist should provide information on the tested sample size and the process that led to the decision to include a sample of such size.

## 3. Results

### 3.1. Position Random Walk Analysis

**Figure 7:**
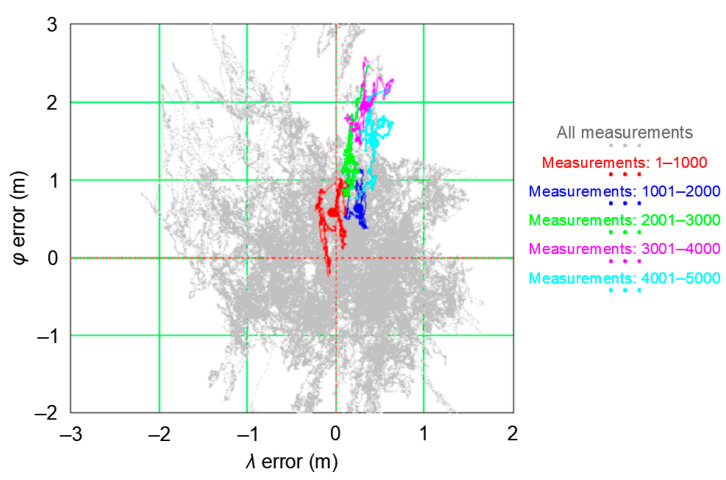
GPS position error distribution during the measurement campaign in 2013.

Let us analyze the PRW process using the GPS system as an example. A two-day measurement session was used for the analysis, carried out with a frequency of 1 Hz by a stationary receiver. The research were carried out at the point with coordinates: *φ* = 54°32.585029′ N and *λ* = 18°32.741505′ E (Poland), where 168′286 positions were recorded on 12–13 March 2013. To analyze the coordinate changes resulting from the Position Random Walk, five smaller sessions with a fixed length of 1000 measurements were distinguished from the whole measurement session. The first session consisted of 1000 initial measurements (measurements: 1–1000), the second session consisted of subsequent 1000 measurements (measurements: 1001–2000), etc., up to the fifth session with measurements: 4001–5000. Figure 7 shows, against the background of the whole session, the position error distributions from all five analyzed sessions, with 1000 measurements each. The position distribution was made in relation to mean values ($\bar{\varphi }$ and $\bar{\lambda }$) calculated for the whole campaign (168′826 measurements). They were marked as the beginning of the coordinate system (0, 0). Additionally, the results of each of the five sessions were marked with different colors. The red color indicates the results of session 1 (measurements: 1–1000), blue indicates the results of session 2 (measurements: 1001–2000), green indicates the results of session 3 (measurements: 2001–3000), purple indicates the results of session 4 (measurements: 3001–4000), and sky-blue indicates the results of session 5 (measurements: 4001–5000).

From the graph, it is clear that the determined position does not “*jump*” randomly around the mean coordinate values ($\bar{\varphi }$ and $\bar{\lambda }$) but it “*walks*”. This feature will be called the “*Position Random Walk*”. Since the measurement results of each of the five sessions are not random, this means that the *φ* and *λ* measurement errors are not, as assumed, normally distributed.

The obvious questions that arise here are:

1. How fast (in terms of changes in coordinates between successive measurements) is the PRW process?
2. Does the Position Random Walk process depend on the nominal (typical) navigation system position error?

It is advisable to introduce the following markings, which will facilitate referencing the research, for numerical analysis of this phenomenon based on the example of GPS system:

- ${\bar{\mu }}_{E}\left(\varphi_{GPS},S_{N}\right)$—$\bar{\varphi }$ calculated from empirical GPS data for the session *S* number *N*, for *N =* 1, 2, 3, 4, 5. Each session consists of 1000 measurements. The session is part of the entire campaign of 168′286 measurements.
- *μ_T_(φ_GPS_)*—nominal (assumed to be real) value of *φ* of the measuring receiver typical for the GPS system. In the analyses, it is determined as an arithmetic mean of *φ* from empirical measurements from a very long measurement campaign. Its results should be very close to the real values, so they were considered to be true (reference) for the GPS system.
- ${\bar{\mu }}_{E}\left(\lambda_{GPS},S_{N}\right)$—$\bar{\lambda }$ calculated from empirical GPS data for the session *S* number *N*, for *N =* 1, 2, 3, 4, 5. Each session consists of 1000 measurements. The session is part of the entire campaign of 168′286 measurements.
- *μ_T_(λ_GPS_)*—nominal (assumed to be real) value of *λ* of the measuring receiver typical for the GPS system. In the analyses, it is determined as an arithmetic mean of *λ* from empirical measurements from a very long measurement campaign. Its results should be very close to the real values, so they were considered to be true (reference) for the GPS system.
- $\bar{P}\left(S_{N}\right)$—mean 2D position calculated from empirical GPS data for the session *S* number *N*, for *N = 1,2,3,4,5*, with the following values: ${\bar{\mu }}_{E}\left(\varphi_{GPS},S_{N}\right)$ and ${\bar{\mu }}_{E}\left(\lambda_{GPS},S_{N}\right)$. Each session consists of 1000 measurements. The session is part of the entire campaign of 168′286 measurements.
- *δ_E_(φ_GPS_,S_N_)*—RMS with respect to $\bar{\varphi }$ calculated from empirical GPS data for the session *S* number *N*, for *N =* 1, 2, 3, 4, 5.
- *δ_T_(φ_GPS_)*—nominal (assumed to be real) value of RMS with respect to $\bar{\varphi }$. It was calculated based on GPS empirical data from a very long measurement campaign (168′286 measurements) and was therefore considered true (reference) for the GPS system.
- *δ_E_(λ_GPS_,S_N_)*—RMS with respect to $\bar{\lambda }$ calculated from empirical GPS data for the session *S* number *N*, for *N =* 1, 2, 3, 4, 5.
- *δ_T_(λ_GPS_)*—nominal (assumed to be real) value of RMS with respect to $\bar{\lambda }$. It was calculated based on GPS empirical data from a very long measurement campaign (168′286 measurements) and was therefore considered true (reference) for the GPS system.

In order to assess the speed of the GPS coordinate shift (PRW process), each session was analyzed separately. The distance *d(S_K_)* calculated between the mean 2D coordinates from subsequent sessions (1000 measurements each) using the following formula was considered as a measure of the PRW process speed:

(8) $$d\left(S_{K}\right)=\left[\bar{P}\left(S_{K+1}\right)-\bar{P}\left(S_{K}\right)\right]=\sqrt{{\left[{\bar{\mu }}_{E}\left(\varphi_{GPS},S_{K+1}\right)-{\bar{\mu }}_{E}\left(\varphi_{GPS},S_{K}\right)\right]}^{2}+{\left[{\bar{\mu }}_{E}\left(\lambda_{GPS},S_{K+1}\right)-{\bar{\mu }}_{E}\left(\lambda_{GPS},S_{K}\right)\right]}^{2}}$$

where

*d(S_K_)*—distance between the mean 2D coordinates for the session *S_K+_*_1_ and *S_K_*, for *K =* 1, 2, 3, 4.

**Figure 8:**
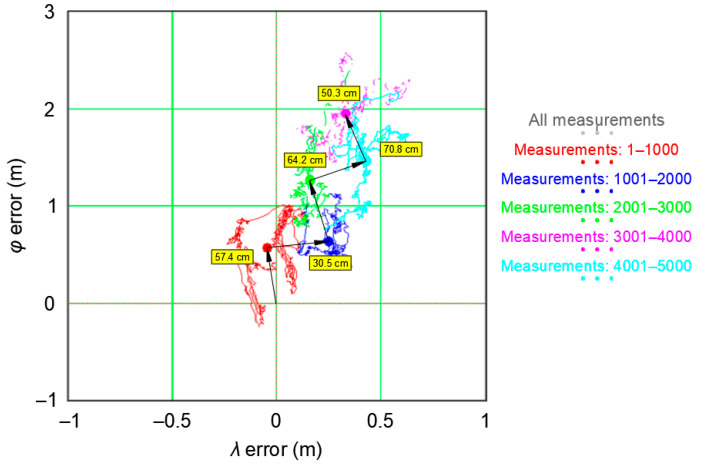
Distances between the mean coordinates $\bar{P}\left(S_{N}\right)$ calculated for five GPS sessions during the measurement campaign in 2013.

Figure 8 presents the values of *d(S_K_)* for five sessions with numbers *N*. This resulted in four distances numbered *K*. Moreover, the drawing shows the distance between the center of the coordinate system and the mean position from the first session. The positions $\bar{P}\left(S_{N}\right)$ are marked with large dots and the dot colour corresponds to the session $S_{N}$.

Figure 8 shows that the mean coordinates calculated for the session 1 $\left[\bar{P}\left(S_{1}\right)\right]$, marked in red, are 57.4 cm away from the mean coordinates from the whole campaign (0, 0). The mean coordinates of the subsequent session 2 $\left[\bar{P}\left(S_{2}\right)\right]$, marked in blue, are 30.5 cm away from the mean coordinates from the session 1 $\left[\bar{P}\left(S_{1}\right)\right]$, marked in red. The subsequent mean coordinates and distances between them have been marked in an analogue manner. The arrows in Figure 8 indicate the movement direction of the position $\bar{P}\left(S_{N}\right)$, with the distances *d(S_K_)* being marked next to them.

The PRW has been shown to influence the mean position [$\bar{P}\left(S_{N}\right)$ calculated from a short 1000 measurement session], as well as the ${\bar{\mu }}_{E}\left(\varphi_{GPS},S_{N}\right)$ and ${\bar{\mu }}_{E}\left(\lambda_{GPS},S_{N}\right)$ determined from the same sample. If we assume that, based on the recording of 1000 GPS measurements, we attempt to assess its accuracy and draw conclusions about the dispersion of measurement results, it is reasonable to ask whether or not a sample of this length is representative. To do so, let us compare the results from five empirical sessions (1000 measurements) with the results from the whole campaign (168′286 measurements), which can be considered close to the real values (Table 3).

When assessing the $\bar{\varphi }$ shift for the whole campaign, $\mu_{T}\left(\varphi_{GPS}\right)-{\bar{\mu }}_{E}\left(\varphi_{GPS},S_{N}\right)$, the value of 1.956 m from the session 4 is immediately noticeable. This almost doubles the standard deviation from the whole campaign. Therefore, this result should be considered as an outlier error. Longitude was determined much more precisely. The offset $\mu_{T}\left(\lambda_{GPS}\right)-{\bar{\mu }}_{E}\left(\lambda_{GPS},S_{N}\right)$ is 0.223 m on average, while the shift $\mu_{T}\left(\varphi_{GPS}\right)-{\bar{\mu }}_{E}\left(\varphi_{GPS},S_{N}\right)$ is as much as 1.178 m.

Please note that the standard deviations of position errors, calculated independently for session coordinates consisting of 1000 measurements, are much lower than those determined from the entire population (Table 3). Therefore, we can conclude that the measurement sessions significantly overestimate the actual accuracy of the tested GPS system.

The reliability in calculating the values of *δ_E_(φ_GPS_,S_N_)* and *δ_E_(λ_GPS_,S_N_)* is crucial. These are values identical to the 2D position error of the navigation system determined with a probability of *p* = 0.68 with the following relationship:

(9) $$DRMS\left(p=0.68\right)=\sqrt{\delta_{E}{\left(\varphi_{GPS},S_{N}\right)}^{2}+\delta_{E}{\left(\lambda_{GPS},S_{N}\right)}^{2}}$$

The analysis of the values of *δ_E_(φ_GPS_,S_N_)* and *δ_E_(λ_GPS_,S_N_)* shows that if these values are overestimated by 65.97% (*φ*) and 86.89% (*λ*), then DRMS(*p* = 0.68) is overestimated by:

(10) $$DRMS\left(p=0.68\right)=\sqrt{{\left(65.97\%\right)}^{2}+{\left(86.89\%\right)}^{2}}=109.1\%$$

That is 109.1%. Therefore, a GPS measurement session aimed to determine its accuracy cannot be considered representative.

The data presented in Table 3 clearly indicate that the sample of 1000 GPS measurements is unrepresentative for this system due to the significant differences between the standard deviations of *φ* and *λ* measurements for all 1000 measurements sessions and measurements from the whole campaign. Furthermore, the overestimation of the *δ_E_(φ_GPS_,S_N_)* value reaches 65.97%, while the *δ_E_(λ_GPS_,S_N_)* value is overestimated by as much as 86.89%. This means that inferences on position error will be burdened with a large error.

Another question that arises is whether this is the case in other positioning systems and how large is the mean position offset for 1000 measurements. Two EGNOS systems were selected for analysis, as they contained nearly 1 million measurements made at the reference point (real coordinates were known, they were determined by surveying methods with an error of about 2 cm with a probability of 95%). Moreover, for PRW analyses, measurements of Decca Navigator system from 1993 were used. This non-existent positioning system was chosen because, unlike most systems operating today which measure distances, the Decca Navigator system determines the distance differences; hence, its principle of operation is significantly different. An analysis of the Decca Navigator system’s errors will answer the question of whether the Position Random Walk was also characteristic for this system.

**Figure 9:**
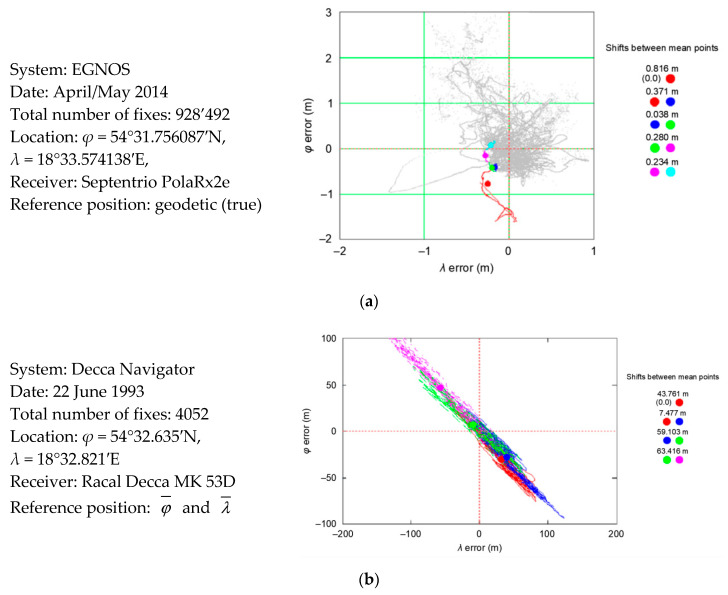
PRW process based on the example of (**a**) EGNOS; (**b**) Decca Navigator.

The EGNOS system was also deliberately selected because its 2D position error is almost twice as small as the 2D position error of the GPS system. This allows the answer to the second question: does the PRW process depend on the nominal (typical) navigation system position error. Figure 9 presents the phenomenon of Decca Navigator and EGNOS Position Random Walk, together with the values of mean position shifts from sessions of 1000 measurements. This is identical to an analysis of the PRW process for the GPS system.

Figure 9a shows that the first 5000 EGNOS measurements were located to the west and south of the mean coordinates determined from the whole population (928′492 fixes). It was similar for GPS measurements, as the process of coordinates walk and mean shift was also confirmed by the measurements of the now non-existent Decca Navigator system made in 1993 (Figure 9b). Compared to GPS and EGNOS systems, the distances between the mean position values from 1000 measurements sessions are much larger. It is clear that as the accuracy of the positioning system increases, the distance between the mean coordinate values from 1000 measurement sessions decreases.

Table 4 summarizes the values of mean position coordinates offsets between 1000 measurement sessions for three navigation positioning systems: GPS, EGNOS and Decca Navigator. The second column contains the R95 value calculated for the whole population (168′286 measurements). This indicates the nominal accuracy of the positioning system. Subsequent column presents values of the mean position coordinates shift between sessions 1 and 2. The fourth column presents offsets between sessions 2 and 3, etc. The mean value of shifts counted from all sessions can be read in column 7. This value proves that the more accurate the positioning system, the smaller the mean PRW observed.

Based on data presented in Table 4, it is clear that the distance by which the mean 2D position shifts from 1000 measurements (made within 1000 s) varies across the positioning systems and is related to the accuracy of position determination. EGNOS, as a GPS augmentation system, has a much lower error than GPS system, and the observed mean offset of the mean 2D position from 1000 measurements is also lower (23.1 cm) than GPS system (54.0 cm). However, in the Decca Navigator system with significantly lower accuracy, the mean 2D position shift from 1000 measurements is at the level of several dozen meters (43.332 m), which is much more than for GPS and EGNOS systems.

### 3.2. Influence of Position Random Walk on Determining the Navigation System Positioning Accuracy

The PRW phenomenon, observed in GPS system and other positioning systems, absolutely must be considered when an attempt is made to determine the accuracy of any positioning system based on measurements. The assumption that the length of a measurement session, aimed at assessing the accuracy of any positioning system, can be arbitrary is simply not true. There is no doubt, however, that the length of the measurement session (number of measurements) must be representative. This term means that if the length of the measurement session is chosen right, the value of navigation system position error calculated on its basis will be close to the nominal (real) value of the system under analysis. This thesis can be proven, for example, with Figure 7 or Figure 9, in which it is clear that the standard deviation from a single session consisting of 1000 measurements is several times smaller than the RMS deviation for the whole campaign. This gives us the certainty that the assessment of the GPS accuracy of position determination based on a session of 1000 measurements will be false.

**Figure 10:**
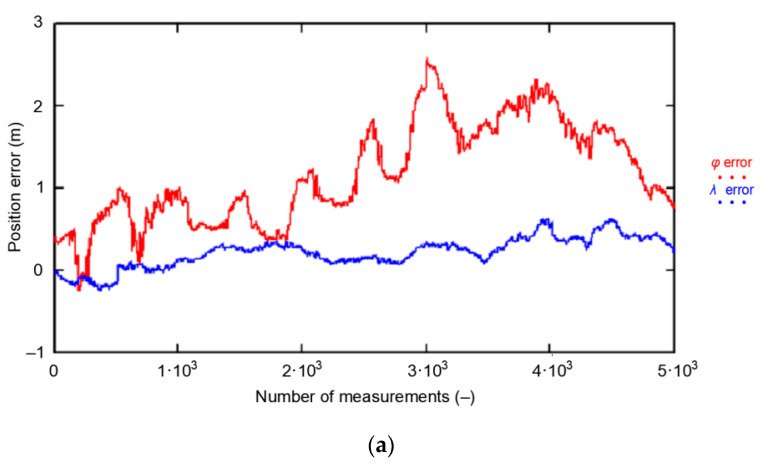

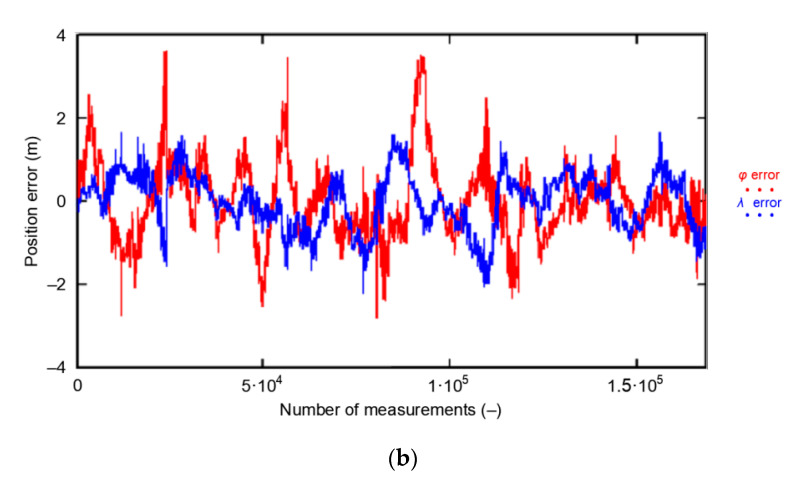
GPS position error for (**a**) 5000; (**b**) 168′286 fixes during the GPS measurement campaign in 2013.

In analyzing the results from the GPS measurement campaign in 2013 in more detail, it should be noted that out of the first 5000 positions (from a sample of 168′286 measurements), there are practically no measurements to the west of the mean coordinates (0, 0) calculated from the whole population (Figure 10a). In addition, the vast majority of the measurements are located north of the mean coordinates. This means that the measurements to the south and west of the center of the coordinate system only appear later in the measurement campaign so that the $\bar{\varphi }$ and $\bar{\lambda }$ calculated for the whole population are at S (*φ*) and E (*λ*) from the first 5000 measurements under consideration. This hypothesis is confirmed by Figure 10b, where the *φ* and *λ* measurement errors in the whole population are presented (168′286 measurements). Therefore, a session of even 5000 measurements will not be representative.

The failure to achieve representativeness of each of the five 1000 measurement sessions analyzed mainly relates to the *δ_E_(φ_GPS_,S_N_)* and *δ_E_(λ_GPS_,S_N_)* determined for each session separately. This can clearly be seen in the histograms of *φ* errors for the whole population and the five population fragments consisting of 1000 measurements. The sessions have been color-coded in the same way as in the previous analysis.

**Figure 11:**
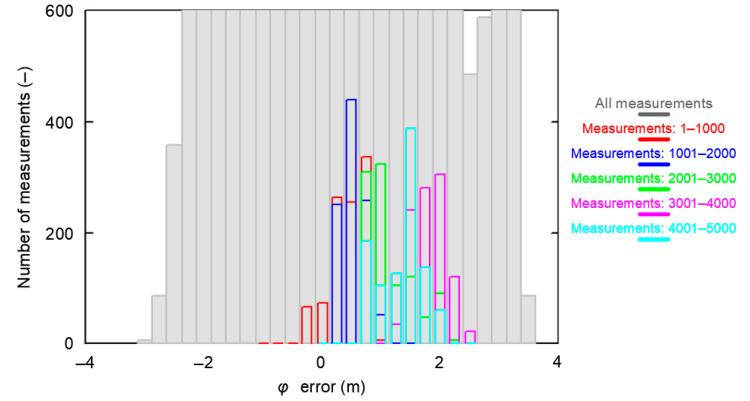
Histogram of GPS *φ* errors determined for the whole population (168′286 fixes, grey) and the five sessions (1000 color-coded measurements each) during the measurement campaign in 2013. The width of a single histogram bar is 0.25 m.

From Figure 11 it is clear that the widths of the histogram bar from sessions *S_N_*, which are closely related to the standard deviations of these samples *δ_E_(φ_GPS_,S_N_)*, are significantly smaller than *δ_T_(φ_GPS_)* for which the results of the whole campaign (grey) were accepted. Therefore, they cannot be considered representative of this system. Even if the results of all five 1000 measurement sessions were combined, the results for a session of even 5000 measurements would still deviate from the representative session in terms of RMS values. Due to the obvious similarities, the results of the *λ* analysis would be very similar.

### 3.3. Representative Sample Size

PRW in navigation positioning systems not only causes the results of short measurements series to be unrepresentative but also calls into question the normality of the distribution of statistical accuracy of *φ* or *λ* errors. Statistical testing for one of the samples *S*_1_ using the non-parametric Kolmogorov–Smirnov statistical test was thus justified. This is the basic and most frequently used test for verifying the fit of empirical data with the normal distribution. The Kolmogorov–Smirnov statistic (*D*) is based on the largest vertical difference between the theoretical (*F_n_*) and the empirical (*F*) cumulative distribution function:

(11) $$D_{n}=\underset{x}{\sup}\left|F_{n}\left(x\right)-F\left(x\right)\right|$$

The null and the alternative hypotheses are: *H*_0_—the data follow the specified distribution and *H_A_*—the data do not follow the specified distribution. The hypothesis regarding the distributional form is rejected at the chosen significance level if the test statistic, *D*, is greater than the critical value obtained from a table. The fixed values of *α* amounting to 0.01, 0.02, 0.05, 0.1, and 0.2 are generally used to evaluate the null hypothesis at various significance levels. A value of 0.05 is typically used for most applications. The *p*-value, in contrast to fixed (*α*) values, is calculated based on the test statistic and denotes the threshold value of the significance level in the sense that the *H*_0_ hypothesis will be accepted for all values of *α* less than the *p*-value. EasyFit software was used for the test. The *φ* and *λ* errors for the GPS session no. 1 were tested. The test results are presented in Table 5, where for both variables (*φ* and *λ* errors in *S*_1_) a graph of empirical and theoretical distribution is presented together with differences between empirical and theoretical distributions and the results of a Kolmogorov–Smirnov test.

Statistical testing of fit for empirical *φ* and *λ* errors with the normal distribution showed that *φ* and *λ* errors for the session *S*_1_ of the GPS system are not normally distributed. For this reason, inferring the accuracy of GPS measurements based on a session of only 1000 measurements also give false results.

A session of 1000 measurements has been shown to be unrepresentative, and Figure 11 seems to demonstrate that a session of 5000 measurements is equally unrepresentative. Therefore, the key question to be asked here is: how long (number of measurements) should a measurement session be for it to be representative? The question is actually equivalent to determining the number of measurements for which:

(12) $${\bar{\mu }}_{E}\left(\varphi_{GPS},S_{n}\right)≅\mu_{T}\left(\varphi_{GPS}\right)$$

(13) $${\bar{\mu }}_{E}\left(\lambda_{GPS},S_{n}\right)≅\mu_{T}\left(\lambda_{GPS}\right)$$

(14) $$\delta_{E}\left(\varphi_{GPS},S_{n}\right)≅\delta_{T}\left(\varphi_{GPS}\right)$$

(15) $$\delta_{E}\left(\lambda_{GPS},S_{n}\right)≅\delta_{T}\left(\lambda_{GPS}\right)$$

This translated into an experiment in which the number of measurements is increased while calculating the RMS. The sought value will be the number of measurements for which the root mean square values are close to the nominal (real) GPS values. The results from the whole campaign of 168′286 measurements being taken as real values. For this purpose, the following calculation experiment was carried out.

1. The whole population of 2D position errors (168′286 measurements) was divided into 168 sessions with the number of measurements increasing by a fixed value (1000 measurements). This means that the length of the first session was 1000 measurements, the subsequent session consisted of 2000 measurements, then 3000 measurements up to a session of 168′000 measurements.
2. For each of the sessions, the ${\bar{\mu }}_{E}$ and *δ_E_* values were determined, followed by the DRMS(2D) value on their basis.
3. Such a session (of minimum length) will be considered representative if the *δ_E_* values is close to the standard deviation from the whole campaign.

**Figure 12:**
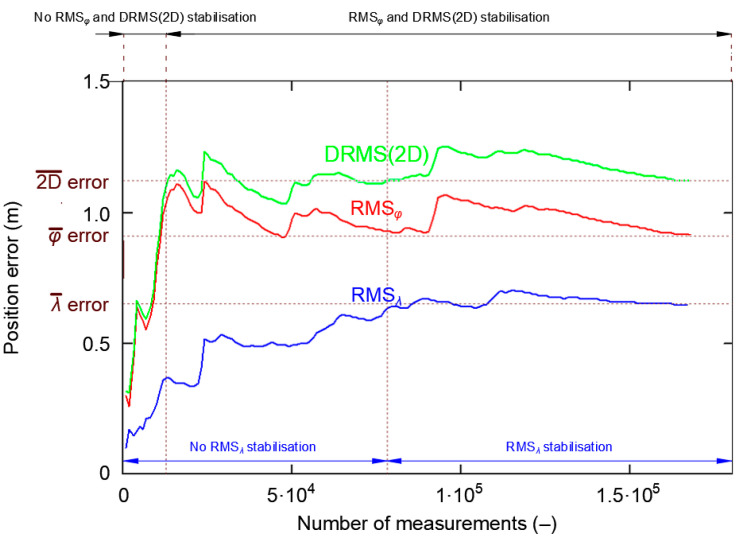
The process of stabilizing Root Mean Square (RMS) values for *φ* and *λ*, as well as Distance Root Mean Square (DRMS)(2D) for 168′000 fixes during the GPS measurement campaign in 2013.

Figure 12 shows the cumulative standard deviation for *φ*, *λ* and DRMS(2D) errors. The session length increases from 1000 to 168′000 measurements, with a step of 1000 measurements. There are two areas to characterize each of the three curves. In the first area, the RMS value increases strongly before achieving stabilization. Latitude RMS and DRMS(2D) stabilize at about 13′000 measurements, while longitude RMS stabilizes at about 78′000 measurements. It can thus be concluded that after 13′000 measurements have been taken, their standard deviation will be close to the actual value, even though the RMS*_λ_* has not yet reached this stability level.

Please note that once RMS curves stabilize, momentary disturbances in their behavior can be found, but these must be differentiated from the process of value stabilization, as these disturbances result from a momentary decrease in the accuracy of the system’s position, which is caused by a change in the Horizontal Dilution of Precision (HDOP) values due to a change in the number of satellites used.

**Figure 13:**
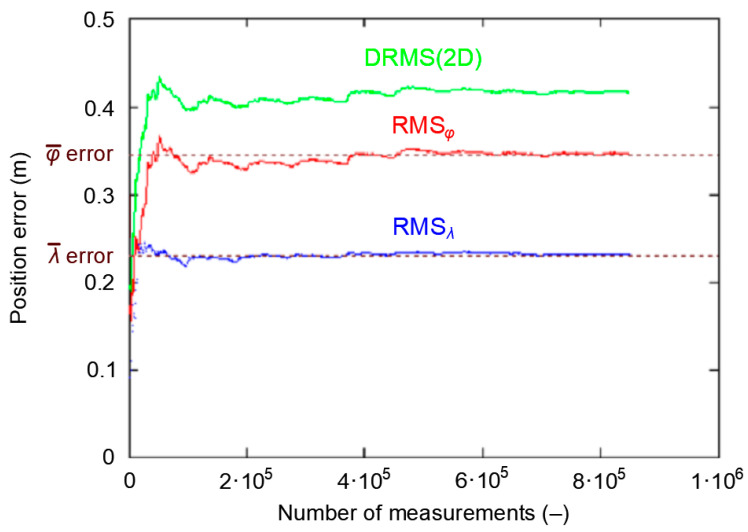
The process of stabilizing RMS values for *φ* and *λ*, as well as DRMS(2D) for 864′000 fixes during the DGPS measurement campaign in 2014.

In order to verify the hypothesis on the existence of two periods (initial instability and stabilization) of the RMS error value, this phenomenon should be assessed against a different positioning system. A nearly one million measurement session of the DGPS system (864′000 fixes) from 2014 was selected for the study. Its length corresponds to a continuous measurement carried out for 10 days at a frequency of 1 Hz. The research were carried out at the point with coordinates: *φ* = 54°31.755238′ N and *λ* = 18°33.574183′ E. The measurements were carried out using the Leica MX9212 receiver. Figure 13 shows root mean square values for *φ* and *λ*, as well as DRMS(2D) for the whole campaign. The graph clearly confirms that the PRW also results in lack of stability and stabilization of RMS values in the DGPS system.

**Figure 14:**
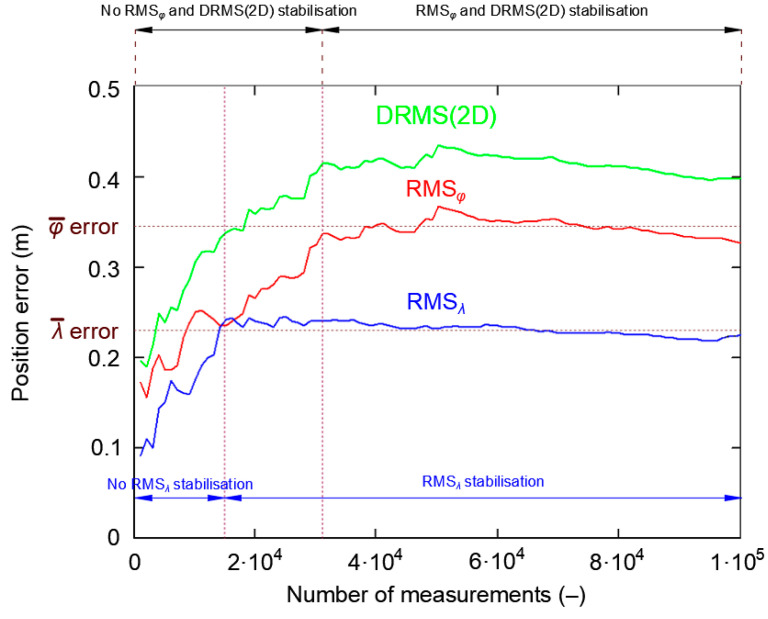
The process of stabilizing RMS values for *φ* and *λ*, as well as DRMS(2D) for the first 100′000 fixes during the DGPS measurement campaign in 2014.

To assess whether stabilization of RMS error values is similar to the GPS system, only the first 100′000 measurements are presented in Figure 14.

Figure 14 confirms that the stabilization process is necessary to obtain a stable RMS error value. For the DGPS system, the RMS*_λ_* stabilized over the first 15′000 measurements. Latitude RMS and DRMS(2D) stabilized at approximately 31′000 measurements. This means that the DGPS measurement sessions, which exceed 31′000 measurements, should be representative and their results will be close to the real measurements.

## 4. Discussion

It has been demonstrated that the PRW phenomenon occurs in systems, such as: GPS, DGPS, EGNOS, and Decca Navigator, which means that, due to the iterative positioning method, it characterizes most navigation positioning systems. For assessing the accuracy of position determination, it has a significant impact both on the determination of $\bar{\varphi }$ and $\bar{\lambda }$ values and on the statistical distributions of both of these values calculated from an unrepresentative population. Moreover, depending on the positioning system, or more precisely on its accuracy, the Position Random Walk has a different shift speed of the mean of 1000 measurements. In more precise systems (such as EGNOS), the offset is “*slower*”, while it is faster in systems with lower accuracy.

Determination of the $\bar{\varphi }$ and $\bar{\lambda }$ values from an unrepresentative sample (in the case of GPS system it was 1000 measurements) can lead to significant discrepancies between these coordinates and actual values. The analysis of the five unrepresentative GPS sessions of 1000 measurements showed that the $\bar{\varphi }$ and $\bar{\lambda }$ coordinates were offset by 1.178 m and 0.223 m, respectively. These values should be evaluated in comparison with the actual standard deviations of both of these values (assumed to be the results from the 168′826 measurements campaign), which are: *δ_T_(φ_GPS_)* = 0.910 m and *δ_T_(λ_GPS_)* = 0.653 m. It follows that the *φ* value calculated as an average of the unrepresentative sample is an outlier.

For small samples, another feature of the PRW phenomenon is that *φ* and *λ* errors do not fit the normal distribution. Thus, to calculate the characteristic function for the normal distribution of position accuracy measures (RMS and 2DRMS), we should verify whether the tested sample is representative. From the author’s previous research, it follows that for the GPS system this condition will be met for a sample of about 13′000 measurements, whereas for DGPS it will be met for a sample of about 31′000 measurements. For such a number, the sample statistics are close to the actual values. It should be pointed out that the purpose of this paper was not to precisely define the minimum representative sample size. The paper stresses that Position Random Walk causes problems with the representativeness of results related to the analysis of position accuracy measures. Precise determination of the moment when RMS (*φ* and *λ*) and DRMS(2D) values stabilize constitutes a separate research question and was not discussed in this publication.

In this paper, a number of empirical measurements from positioning systems carried out over 20 years (1993–2014) were used. The analyses required real accuracy of positioning systems, which were not known. Therefore, the actual values of *φ* and *λ*, as well as their standard deviations, were based on results from very large samples. It can be assumed that the results from such large samples are very close to the real accuracy characteristics of these systems. It should also be noted that the accuracy of positioning systems is constantly changing. Constant technical progress results in an increase in the accuracy of the GPS and related systems (DGPS and EGNOS); hence, the values for which the RMS and DRMS values stabilize today will be probably different from those calculated in this paper. For any positioning system, a very long (e.g., one week) measurement campaign is needed to determine them.

Positioning system operators do not publish up-to-date accuracy data regularly. For example, the official document on GPS system, the most widely used satellite navigation system in the world, has only been published once over the last decade. This paper was entitled “*Global Positioning System Standard Positioning Service Signal Specification*”. The same is true of the other positioning systems (GLONASS, BeiDou Navigation Satellite System (BDS), Galileo, DGPS, EGNOS, and others), as well as multi-Global Navigation Satellite System (GNSS) solutions (GPS/GLONASS, GPS/GLONASS/BDS, GPS/GLONASS/Galileo, and GPS/GLONASS/BDS/Galileo) whose current positioning accuracy is not precisely known.

Knowledge of the current accuracy of the positioning system is extremely important as it determines the possible navigation application of the system. With this in mind, researchers often try to assess the accuracy of positioning systems in measurement experiments. The studies referred to in this paper clearly indicate that such an experiment must account for the PRW phenomenon and that sample used should be representative. An example of five sessions of 1000 measurements shows that the accuracy assessment results for a session of this length differ significantly from the actual values. In this case, the DRMS error is overestimated by as much as 109.1%. This is a very important conclusion from this publication.

## 5. Conclusions

The analyses showed that all researched positioning systems (GPS, DGPS, EGNOS, and Decca Navigator) are characterized by the PRW, and *φ* and *λ* errors of the determined position do not appear in a random way characteristic for normal distribution, which was demonstrated with negative statistical tests for *φ* and *λ* errors.

It was shown that the size of the PRW depends on the nominal accuracy of position determination by the system and that for systems with lower accuracy (Decca Navigator), the coordinate change in subsequent measurements is greater than for systems with higher accuracy (EGNOS).

Research has shown that to be able to assess the accuracy of a positioning system, it is necessary to correctly determine the length of the measurement session (number of measurements). Based on the analysis of GPS system, it follows that sessions consisting of 1000 measurements significantly overestimate the results and their statistics must not be considered real values.

This article discusses the problem of determining a representative length of the measurement session for positioning systems, resulting from the PRW process. Its essence consists in finding a point of stabilization of the RMS (*φ* and *λ*) and DRMS(2D) values. Selected examples (GPS and DGPS) show that a minimum length of a representative measurement session is needed to assess the accuracy. It should be noted that the issue of determining the point beyond which the RMS and DRMS values stabilize requires further research. Furthermore, the position of this point will be constantly changing due to the continuous development and improvement of positioning systems.

The article begins a series of monothematic publications, the aim of which will be statistical distribution analysis of navigation positioning system errors. One of the next research issues that has not been studied in this article will be the determination of factors that influence changes in the PRW phenomenon. Apart from three hypotheses (the iterative method used to determine coordinates, where the next position is calculated from the previous one, widespread use of Kalman filtering in positioning system receivers for filtering out movement parameters and normally navigation positioning system errors are correlated over time) mentioned in the introduction, it is also planned to determine the impact of GNSS errors (ionospheric and tropospheric effects, multipath, noise, etc.) on the Position Random Walk.

## Figures and Tables

**Table 1:** Relationship between the population and probability in normal as well as chi-squared distributions.

Normal Distribution			Chi-Squared Distribution		
α	Probability (%)	Notation	$\sqrt{\alpha }$	Probability (%)	Notation
0.67	50.0	LEP	1.00	39.4	1σ level or standard ellipse
1.00	68.3	1σ level or RMS	1.18	50.0	CEP
1.96	95.0	95% confidence level	$\sqrt{2}$	63.2	DRMS
2.00	95.4	2σ level	2.00	86.5	2σ ellipse
3.00	99.7	3σ level	2.45	95.0	95% confidence level
			$2\sqrt{2}$	98.2	2DRMS
			3.00	98.9	3σ ellipse

**Table 2:** Allowable decisions when testing statistical hypotheses.

		Reality	
		*H*_0_	*H*_1_
Decision	*H*_0_	Correctly accepted *H*_0_ (1−*β*)	Type II error (*β*)
	*H*_1_	Type I error (*α*)	Correctly rejected *H*_0_ (1−*α*)

**Table 3:** Shift values in measurement results (*φ* and *λ*) and RMS values of five sessions of 1000 measurements each, compared to the whole population (168′286 fixes) during the GPS measurement campaign in 2013.

Measure	All Fixes (168′286)	*S*_1_—Red Session (1–1000)	S_2_—Blue Session (1001–2000)	S_3_—Green Session (2001–3000)	S_4_—Purple Session (3001–4000)	S_5_—Sky-Blue Session (4001–5000)	Mean from S_1_–S_5_
$\bar{\varphi }$ shift $\mu_{T}\left(\varphi_{GPS}\right)-{\bar{\mu }}_{E}\left(\varphi_{GPS},S_{N}\right)$	0.000 m	0.572 m	0.633 m	1.268 m	1.956 m	1.463 m	1.178 m
$\bar{\lambda }$ shift $\mu_{T}\left(\lambda_{GPS}\right)-{\bar{\mu }}_{E}\left(\lambda_{GPS},S_{N}\right)$	0.000 m	−0.047 m	0.251 m	0.157 m	0.326 m	0.427 m	0.223 m
Distance of point $\bar{P}\left(S_{N}\right)$ from the real coordinates (0, 0)	0.000 m	0.574 m	0.681 m	1.278 m	1.983 m	1.524 m	1.208 m
$\delta_{E}\left(\varphi_{GPS},S_{N}\right)$	0.910 m	0.299 m	0.201 m	0.414 m	0.275 m	0.358 m	0.309 m
Session RMS*φ* vs all measurements RMS*φ* (%) $\frac{\delta_{E}\left(\varphi_{GPS},S_{N}\right)}{\delta_{T}\left(\varphi_{GPS}\right)}\cdot 100\%$	100.00%	32.86%	22.12%	45.54%	30.26%	39.37%	34.03%
$\delta_{E}\left(\lambda_{GPS},S_{N}\right)$	0.653 m	0.098 m	0.066 m	0.059 m	0.120 m	0.085 m	0.086 m
Session RMS*_λ_* vs all measurements RMS*_λ_* (%) $\frac{\delta_{E}\left(\lambda_{GPS},S_{N}\right)}{\delta_{T}\left(\lambda_{GPS}\right)}\cdot 100\%$	100.00%	15.07%	10.09%	9.01%	18.36%	13.01%	13.11%

**Table 4:** Position coordinates shifts between sessions for three navigation positioning systems: GPS, EGNOS and Decca Navigator.

Positioning System	Empirical Accuracy of All Fixes (R95)	*P*_1_(1–1000) vs. *P*_2_(1001–2000)	*P*_2_(1001–2000) vs. *P*_3_(2001–3000)	*P*_3_(2001–3000) vs. *P*_4_(3001–4000)	*P*_4_(3001–4000) vs. *P*_5_(4001–5000)	Mean Shift between Sessions
GPS	2.039 m	30.5 cm	64.2 cm	70.8 cm	50.3 cm	54.0 cm
EGNOS	0.871 m	37.1 cm	3.8 cm	28.0 cm	23.4 cm	23.1 cm
Decca Navigator	131.719 m	7.477 m	59.103 m	63.416 m	No data	43.332 m

**Table 5:** Statistical analysis of *φ* and λ for the GPS session no. 1 during the measurement campaign in 2013.

Probability density function for *φ* error	
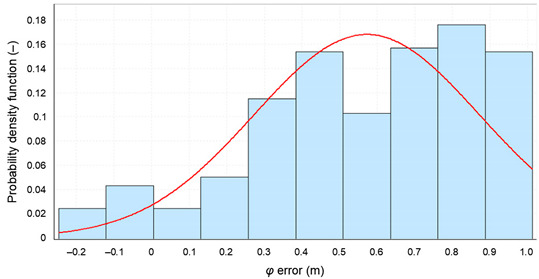	
Probability density function for *λ* error	
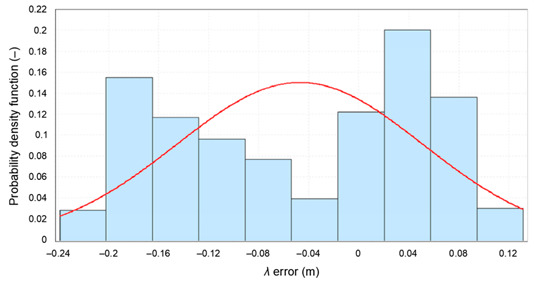	
Probability difference for *φ* error	
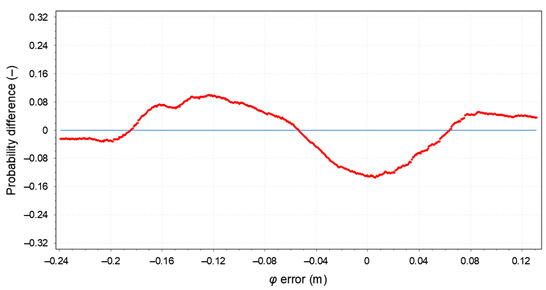	
Probability difference for *λ* error	
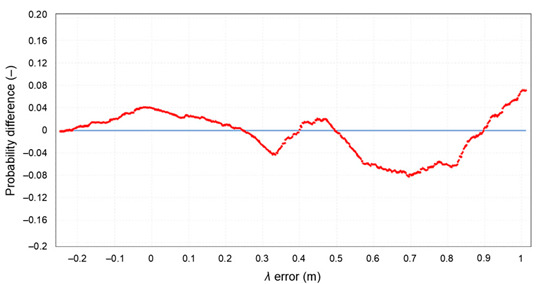	
Kolmogorov–Smirnov test	
*φ* error	*λ* error
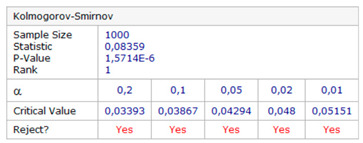	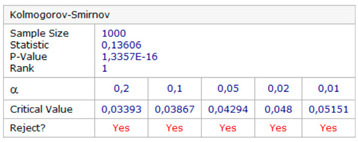

## References

[1] Bowditch N. (2019). American Practical Navigator.

[2] Hofmann-Wellenhof B., Legat K., Wieser M. (2003). Navigation—Principles of Positioning and Guidance.

[3] Specht C. (2007). System GPS.

[4] IALA AISM Navigation. https://www.iala-aism.org/wiki/dictionary/index.php/Navigation.

[5] Cutler T.J. (2003). Dutton’s Nautical Navigation.

[6] Krasuski K., Ćwiklak J., Jafernik H. (2018). Aircraft Positioning Using PPP Method in GLONASS System. Aircr. Eng. Aerosp. Technol..

[7] Marais J., Beugin J., Berbineau M. (2017). A Survey of GNSS-based Research and Developments for the European Railway Signaling. IEEE Trans. Intell. Transp. Syst..

[8] Ambroziak S.J., Katulski R.J., Sadowski J., Siwicki W., Stefański J. (2011). Ground-based, Hyperbolic Radiolocation System with Spread Spectrum Signal—AEGIR. TransNav Int. J. Mar. Navig. Saf. Sea Transp..

[9] Chen Q., Niu X., Zuo L., Zhang T., Xiao F., Liu Y., Liu J. (2018). A Railway Track Geometry Measuring Trolley System Based on Aided INS. Sensors.

[10] Paziewski J., Sieradzki R., Baryla R. (2018). Multi-GNSS High-rate RTK, PPP and Novel Direct Phase Observation Processing Method: Application to Precise Dynamic Displacement Detection. Meas. Sci. Technol..

[11] Sánchez A., Bravo J.L., González A. (2017). Estimating the Accuracy of Track-surveying Trolley Measurements for Railway Maintenance Planning. J. Surv. Eng..

[12] Dziewicki M., Specht C. (2009). Position Accuracy Evaluation of the Modernized Polish DGPS. Pol. Marit. Res..

[13] Specht C., Pawelski J., Smolarek L., Specht M., Dąbrowski P. (2019). Assessment of the Positioning Accuracy of DGPS and EGNOS Systems in the Bay of Gdansk Using Maritime Dynamic Measurements. J. Navig..

[14] Bowditch N. (1984). American Practical Navigator: An Epitome of Navigation.

[15] Langley R.B. (1991). The Mathematics of GPS. GPS World.

[16] van Diggelen F. (1998). GPS Accuracy: Lies, Damn Lies, and Statistics. GPS World.

[17] U.S. DoD (1993). Global Positioning System Standard Positioning Service Signal Specification.

[18] Specht C. Preliminary Accuracy Results of EGNOS After the Implementation of Operational Status. Proceedings of the 5th International Conference & Exhibition (MELAHA 2010).

[19] Pearson K. (1905). The Problem of the Random Walk. Nature.

[20] Mertikas S.P. Error Distributions and Accuracy Measures in Navigation: An Overview. https://unbscholar.lib.unb.ca/islandora/object/unbscholar%3A8708.

[21] Kendall M.G., Buckland W.R. (1982). A Dictionary of Statistical Terms.

[22] Feller W. (1968). An Introduction to Probability Theory and Its Applications.

[23] Biecek P. Wybrane Testy Normalności. http://tofesi.mimuw.edu.pl/~cogito/smarterpoland/samouczki/testyNormalnosci/testyNormalnosci.pdf.

[24] Anderson T.W., Darling D.A. (1954). A Test of Goodness of Fit. J. Am. Stat. Assoc..

[25] Cramér H. (1928). On the Composition of Elementary Errors. Scand. Actuar. J..

[26] von Mises R.E. (1928). Wahrscheinlichkeit, Statistik und Wahrheit.

[27] Kolmogorov A. (1933). Sulla Determinazione Empirica di una Legge di Distribuzione. G. dell’Istituto Ital. degli Attuari.

[28] Smirnov N. (1948). Table for Estimating the Goodness of Fit of Empirical Distributions. Ann. Math. Stat..

[29] Lilliefors H.W. (1967). On the Kolmogorov-Smirnov Test for Normality with Mean and Variance Unknown. J. Am. Stat. Assoc..

[30] Shapiro S.S., Francia R. (1972). An Approximate Analysis of Variance Test for Normality. J. Am. Stat. Assoc..

[31] Shapiro S.S., Wilk M.B. (1965). An Analysis of Variance Test for Normality (Complete Samples). Biometrika.

[32] Pearson K. (1900). On the Criterion that a Given System of Deviations from the Probable in the Case of a Correlated System of Variables is Such that it Can be Reasonably Supposed to have Arisen from Random Sampling. Lond. Edinb. Dublin Philos. Mag. J. Sci..

[33] Anscombe F.J., Glynn W.J. (1983). Distribution of the Kurtosis Statistic b_2_ for Normal Samples. Biometrika.

[34] D’Agostino R.B. (1970). Transformation to Normality of the Null Distribution of g_1_. Biometrika.

[35] D’Agostino R., Pearson E.S. (1973). Tests for Departure from Normality. Empirical Results for the Distributions of b^2^ and √b^1^. Biometrika.

[36] Masereka E.M., Otieno F.A.O., Ochieng G.M., Snyman J. (2015). Best Fit and Selection of Probability Distribution Models for Frequency Analysis of Extreme Mean Annual Rainfall Events. Int. J. Eng. Res. Dev..

[37] Das S., Mitra K., Mandal M. (2016). Sample Size Calculation: Basic Principles. Indian J. Anaesth..

[38] Górecki T. (2011). Podstawy Statystyki z Przykładami w R.

[39] Kelley K., Preacher K.J. (2012). On Effect Size. Psychol. Methods.

[40] Hedges L.V., Pigott T.D. (2001). The Power of Statistical Tests in Meta-analysis. Psychol. Methods.

[41] Cohen J. (1988). Statistical Power Analysis for the Behavioral Sciences.

[42] Cohen J. (1992). A Power Primer. Psychol. Bull..

[43] Teunissen P.J.G. (1999). The Probability Distribution of the GPS Baseline for a Class of Integer Ambiguity Estimators. J. Geod..

[44] Cohen J. (1962). The Statistical Power of Abnormal-social Psychological Research: A Review. J. Abnorm. Soc. Psychol..

[45] Rossi J.S. (1990). Statistical Power of Psychological Research: What Have We Gained in 20 Years?. J. Consult. Clin. Psychol..

[46] Sedlmeier R., Gigerenzer G. (1989). Do Studies of Statistical Power Have an Effect on the Power of Studies?. Psychol. Bull..

[47] Moher D., Dulberg C.S., Wells G.A. (1994). Statistical Power, Sample Size, and Their Reporting in Randomized Controlled Trials. JAMA J. Am. Med Assoc..

[48] King B.M., Minium E.W. (2009). Statystyka dla Psychologów i Pedagogów.

[49] Cohen J., Wolmann B.B. (1965). Some Statistical Issues in Psychological Research. Handbook of Clinical Psychology.

[50] Wilkinson L., Task Force on Statistical Inference (1999). Statistical Methods in Psychology Journals. Guidelines and Explanations. Am. Psychol..

